# Effects of Delayed Metamorphosis on Larval Survival, Metamorphosis, and Juvenile Performance of Four Closely Related Species of Tropical Sea Urchins (Genus *Echinometra*)

**DOI:** 10.1155/2014/918028

**Published:** 2014-01-27

**Authors:** M. Aminur Rahman, Fatimah Md. Yusoff, A. Arshad, Tsuyoshi Uehara

**Affiliations:** ^1^Laboratory of Marine Biotechnology, Institute of Bioscience, Universiti Putra Malaysia, 43400 UPM Serdang, Selangor, Malaysia; ^2^Department of Aquaculture, Faculty of Agriculture, Universiti Putra Malaysia, 43400 UPM Serdang, Selangor, Malaysia; ^3^Department of Chemistry, Biology and Marine Science, Faculty of Science, University of the Ryukyus, 1 Senbaru, Nishihara-cho, Okinawa 903-0213, Japan

## Abstract

We report here, the effects of extended competency on larval survival, metamorphosis, and postlarval juvenile growth of four closely related species of tropical sea urchins, *Echinometra* sp. A (Ea), *E. mathaei* (Em), *Echinometra* sp. C (Ec), and *E. oblonga* (Eo). Planktotrophic larvae of all four species fed on cultured phytoplankton (*Chaetoceros gracilis*) attained metamorphic competence within 22–24 days after fertilization. Competent larvae were forced to delay metamorphosis for up to 5 months by preventing them from settling in culture bottles with continuous stirring on a set of 10 rpm rotating rollers and larval survival per monthly intervals was recorded. Larval survival was highest at 24 days, when competence was attained (0 delayed period), and there were no significant differences among the four species. Larvae that had experienced a prolonged delay had reduced survival rate, metamorphosis success, and juvenile survival, but among older larvae, Em had the highest success followed by Ea, Eo, and Ec. Juveniles from larvae of all four species that metamorphosed soon after becoming competent tended to have higher growth rates (test diameter and length of spines) than juveniles from larvae that metamorphosed after a prolonged period of competence with progressively slower growth the longer the prolonged period. Despite the adverse effects of delaying metamorphosis on growth parameters, competent larvae of all four species were able to survive up to 5 months and after metamorphosis grew into 1-month-old juveniles in lab condition. Overall, delayed larvae of Em showed significantly higher larval survival, metamorphosis, and juvenile survival than Ea and Eo, while Ec showed the lowest values in these performances. Em has the most widespread distribution of these species ranging from Africa to Hawaii, while Ec probably has the most restricted distribution. Consequently, differences in distribution may be related to differences in the ability to delay metamorphosis.

## 1. Introduction

Planktonic larvae of many marine invertebrates undergo a process of settlement and metamorphosis to establish the bottom dwelling mode of benthic life of the adult. Settlement may be an important factor determining the distribution and abundance of an organism [1–4]. The stimuli necessary for settlement involve a combination of biological, physical, and chemical factors [2, 5–8] including the speed of fluids and contour of the substratum surface [9–11] and luminosity and chemical cues [2, 12–14].

In the absence of an appropriate stimulus, larvae of most marine invertebrates are capable of delaying metamorphosis [9, 15–25]. The length of time that larvae can maintain competence to metamorphose in the absence of an appropriate settlement cue has been the subject of numerous studies [15, 21, 23–31]. Many invertebrates will eventually metamorphose “spontaneously” when metamorphosis is delayed [18, 22, 32]. Speciose marine taxa like echinoderms, molluscs, and polychaete annelids have many species with larvae that retain their competent stage for weeks or months in drifting currents [15, 33, 34] and travel over long distances [35–37]. Recruitment of benthic populations then depends on the numbers of competent larvae transported to the appropriate sites [38]. Availability of competent larvae is affected by biological parameters such as adult reproductive output and reproductive cycles [38, 39], abundance of adult population [40], larval mortality [41], and hydrodynamics such as water current and flow velocity [42, 43]. On large spatial scales, larvae of marine invertebrates in a water column behave as passive particles that are transported to a given site through local current pattern, flow velocity, and particularly near-bottom flow dynamics, which play very important roles in larval retention and settlement [14]. As a byproduct of this discriminatory capability, delaying metamorphosis enhances dispersal potential, which should influence a species' geographic range, the degree of genetic isolation among populations, rates of speciation, and species longevity [15, 44–48].

Delayed metamorphosis can have negative effects on juvenile growth and survival [20–22, 31, 59, 60], time to reproduction [61], and adult fecundity [62]. An effect of delayed metamorphosis on some aspects of postlarval performance has been demonstrated for a wide range of marine invertebrates. These include sponges [60], oysters [63], slipper limpets [27], abalone [64], polychaetes ([20, 62], barnacles [28], crabs [65], bryozoans [61, 66], and sand dollars [16]. However, the effect of delayed metamorphosis on the survival and growth of competent larvae and postmetamorphic juveniles in four closely related tropical sea urchins (genus *Echinometra*) with different geographical ranges has not so far been investigated.

Four genetically and ecologically divergent species of tropical sea urchins belonging to *Echinometra mathaei sensu lato*, species complex occur sympatrically in adjacent microhabitats on Okinawan reef flats, and they are one of the most ubiquitous and abundant shallow water echinoids in the warm Indo-Pacific region [49, 50, 67]. They occur commonly in and around reefs and are widely distributed from central Japan in the north, to southeast Australia in the south, and off Mexico in the east, and to the Gulf of Suez in the west [51, 68]. However, studies on morphology, ecology, allozymes, gamete compatibility, DNA-DNA hybridization, mtDNA, and the loci coding for gamete recognition molecules show at least four independent gene pools of *Echinometra* that exist in the Indo-West Pacific, distinguished as *Echinometra *spp. A, B, C, and D [49, 50, 52–57, 69–71]. Molecular phylogenies indicate *Echinometra* in the central and west Pacific split in the last 1–3 million years [66]. *Echinometra *sp. B is now recognized as *Echinometra mathaei *(Em) [72], while *Echinometra *sp. D belongs in the *Echinometra oblonga* (Eo) species complex, which may include at least three cryptic species [73]. Taxonomic description and designation of the other two species, *Echinometra *sp. A (Ea) and *Echinometra* sp. C (Ec), are yet to be made [53]. The small genetic and morphological differences among the four species coupled with their widespread but different distributions make them a valuable group for studies of marine speciation.

Recent studies have indicated that larvae of *Echinometra* spp. fed on cultured phytoplankton can become competent within 20–24 days after fertilization and will settle and metamorphose in response to crustose coralline algae [51, 54–56, 68]. In this paper, we examined the effect of delaying metamorphosis on larval competence, metamorphosis, and postlarval juvenile survival and growth in four species in the *Echinometra* spp. complex. An additional aim is to understand how delaying metamorphosis might affect the distribution patterns of these diverged taxa throughout the warm Indo-Pacific.

## 2. Materials and Methods

### 2.1. Collection and Spawning

Mature adults of all four species of the *Echinometra mathaei* species complex were collected from the Sesoko coast, Okinawa Island at low tide during their natural breeding season from May to September. Immediately after collection, the specimens were transported to the laboratory at the Department of Chemistry, Biology and Marine Science, University of the Ryukyus, Okinawa, where they were maintained in closed aquaria for no more than 4 days before use. Males and females were then induced to spawn and fertilized following the methods and techniques previously described by Rahman et al. [53, 68].

### 2.2. Larval Rearing

Early stage embryos were reared in standing cultures in small glass beakers. When blastulae were seen swimming at the surface of water, they were transferred to glass bottles, containing 400 mL of sterilized filtered seawater (SFSW), which was stirred constantly on a set of 10 rpm rotating rollers. When the larvae attained the 4-armed pluteus stage, they were reared in the same system (400 or 800 mL glass bottles) with a larval density of 1 individual/mL. All cultures were carried out in SFSW at 26–28°C, approximating ambient water temperature. About 75% of the cultured water was removed by reverse filtration/siphoning every 3 days and replaced with fresh SFSW. Larvae of all four species were supplemented with phytoplankton (*Chaetoceros gracilis*) at concentrations of 1 × 10^4^ and 2 × 10^4^ cells per mL of medium at 4-armed to 6-armed and 8-armed stage periods, respectively [68]. All the larvae reached metamorphic competence within 22–24 days after fertilization.

### 2.3. Induction of Metamorphosis

After 22–24 days of rearing, the full-grown larvae that were deemed competent were used in settlement induction tests. Induction of metamorphosis for all larvae was performed on coralline red algal stones, which were collected from the intertidal zone of Sesoko Island, Okinawa during low tide. Stone sizes were: length 5.5–7.0 cm, width 4.0–5.5 cm, and height 1.5–2.0 cm. Mobile animals and epibionts were removed as far as was possible, and the stones were then thoroughly rinsed with SFSW prior to use in experiments. Experiments were conducted in petri dishes (8.2 × 4.0 cm) containing 60 mL SFSW and a test substratum (algal stones). In each experiment, 12 replicate petri dishes (each with 15 competent larvae) were used per treatment. The status of larvae in experimental petri dishes was determined using a binocular microscope at 24–30 h after initiation of metamorphic induction. Larvae that had attached to the algal substratum with the complete development of adult internal organs as well as the formation of the adult mouth, anus, tubefeet, and spines, were considered to have undergone normal metamorphosis [57].

### 2.4. Juvenile Rearing

The newly metamorphosed juveniles along with their attached algal substratum were transferred on the same day to the aerated small (glass/plastic) aquaria (25 × 20 × 10 cm) in static water condition, and pieces of coralline algal skeletons was added for food. Each treatment was consisted of three replicate aquaria. In all rearing, seawater was partially changed every week with fresh FSW to maintain ambient temperature (26–28°C) and salinity (36 ppt). This was continued for up to one month. Survival of juveniles in each aquarium was determined on a binocular microscope. Juvenile was dislodged from the algal substratum with the help of a needle. Test diameter and length of spines from 15 randomly selected juveniles for each replicate experiments were measured on a dissecting microscope with presetting micrometer.

### 2.5. Effect of Delayed Metamorphosis on Metamorphic Competence and Juvenile Performances

Larvae of all four species developed from eggs fertilized on the same day were reared in 6 replicate culture bottles (400 mL) for each species until 22–24 days following the method and protocol described above. Metamorphic competence was judged on day 24 (0 delayed period) according to the method mentioned above. Over 90% of larvae tested on day 24 attached and metamorphosed within 24–30 h (control). The newly competent larvae were forced to delay metamorphosis for up to 5 month by preventing them from settling in culture bottles by continuous stirring on a set of rotating rollers in clean bottles without inducing substrates, and larval survival per monthly intervals was recorded. Competent larvae were fed on *Chaetoceros gracilis* at a concentration of 2 × 10^4^ cells/mL in weekly intervals, so that nearly all larvae were clearly delaying their metamorphosis as the experiment continued. During each feeding, larvae in these delayed experiments were filtered or pipetted into clean bottles with freshly prepared SFSW weekly to avoid biofilm buildup on the bottle walls. In order to ensure the adequate larval supply for more detailed studies of metamorphosis and juvenile performances, delaying larvae of various ages were maintained in stock culture bottles parallel to the experimental rearing. Induction of metamorphic as well as rearing of juveniles and their measurements were performed following the methods described in previous sections.

### 2.6. Statistical Analysis

Percentage data were arcsine transformed for statistical analyses [74]. This transformation helped to normalize the data and reduced heterogeneity in variances. A “Bartlett's test” was used to analyze the homogeneity of variances. When variances were not significantly heterogeneous and there were no major departures from normality, a one-way analysis of variance (ANOVA) was done followed by Tukey's multiple comparison test. The level for statistical significance was set at 0.05. Untransformed data are presented in the figures.

## 3. Results

### 3.1. Survival of Competent Larvae

**Figure 1:**
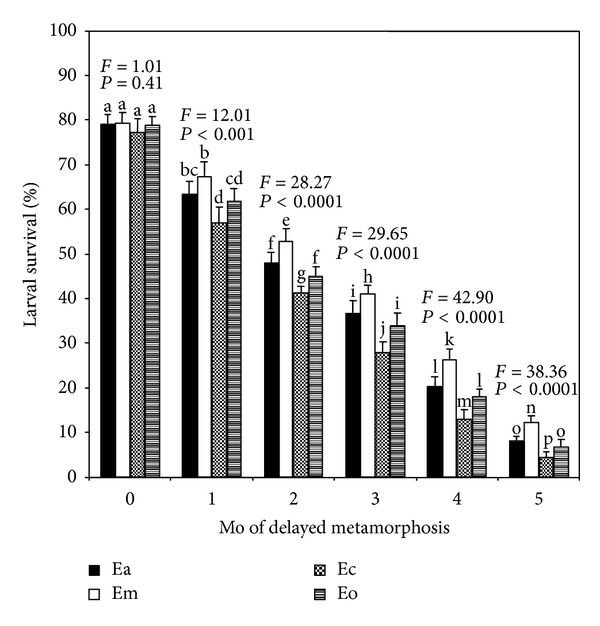
Effects of delayed metamorphosis on larval survival (%) of *Echinometra* sp. A (Ea), *E*.* mathaei* (Em), *Echinometra *sp. C (Ec), and *E*.* oblonga* (Eo). Larvae attained metamorphic competence within 24 days after fertilization were on month 0 (control). Each value represents the mean ± SD of six replicate experiments from each species with 400 larvae per replicate for each delayed period. Results of one-way ANOVA (*F* value and *P* value) are presented above each set of bars. Columns with the same letter represent means that are not significantly different (Tukey's test, *P* > 0.05).

Majority of the larvae of all four species attained the state of metamorphic competence (bottom dwelling mode of life) at 22–24 days after fertilization and was evidenced by the presence of a large adult rudiment and a high percentage of metamorphosis (>90%) in trial assays with coralline red algal covered stones. Larval survival of Em, Ea, Eo, and Ec were highest at 24 days, when competent was attained (0 delayed period), and no significant differences were noted among the four species (Tukey's test, *P* > 0.05) (Figure 1). But the four species differed in survival rates as the delay period increased (Figure 1). One-month delayed larvae of Em and Ea did not differ significantly in survival rates, but Em differed significantly (Tukey's test, *P* < 0.05) from Ec and Eo, while both Ec and Eo did not significantly differ. Moreover, extending the competent phase of larval period 3 to 4 month resulted in striking decreases of survival in a manner that Em exhibited significantly higher (Tukey's test, *P* < 0.05) values followed by Ea/Eo (no differences between Ea and Eo) and Ec in that order (Figure 1).

### 3.2. Metamorphic Success

**Figure 2:**
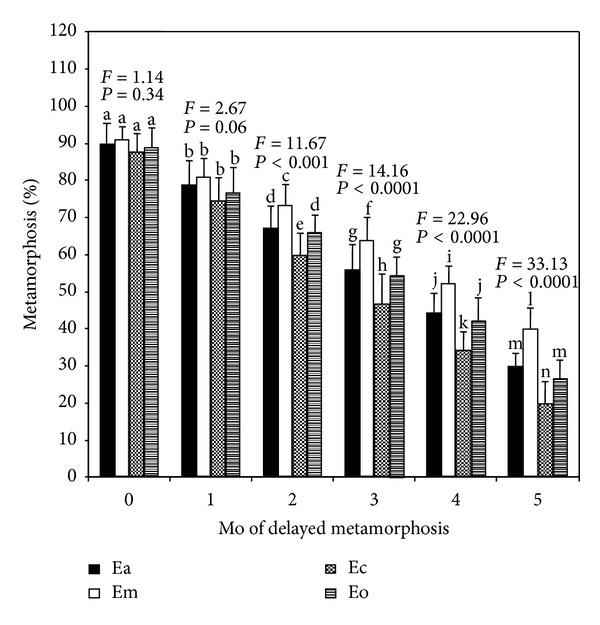
Effects of delayed period on larval competence to metamorphosis (%) of *Echinometra* sp. A (Ea), *E*.* mathaei* (Em), *Echinometra *sp. C (Ec), and *E*.* oblonga* (Eo) in response to coralline red algae. Larvae attained metamorphic competence within 24 days after fertilization were on month 0. Each value represents the mean ± SD of 12 replicate experiments from each species with 15 larvae per replicate for each delayed period. Results of one-way ANOVA (*F* value and *P* value) are presented above each set of bars. Columns with the same letter represent means that are not significantly different (Tukey's test, *P* > 0.05).

Complete metamorphosis from feeding larvae to feeding juveniles took place in about 1 day after settlement on coralline red algal substratum. This included the complete development of internal organs as well as formation of adult mouth, anus, tubefeet, and spines. Completion of metamorphosis was the same and the rate of development was equivalent among the four species. Moreover the time required to complete metamorphosis was similar and no particular deformities/defects were observed in the juveniles among the four species at various delayed periods. The percentage of *Echinometra* spp. larvae that metamorphosed was typically greater for 0 delayed larvae than for delayed larvae whose metamorphosis was postponed (Figure 2). Competent larvae (0 month delayed) of *Echinometra *spp. showed highest metamorphic success and no significant differences (Tukey's test, *P* > 0.05) were recognized among the four species (Figure 2). Similar but significantly lower metamorphosis rate was observed in the metamorphosis of 1-month delayed larvae (Figure 2). Moreover, postponing metamorphosis of 2–5-month delayed larvae had significant effect on metamorphosis. Despite these discriminations, an extended larval period resulted in lower metamorphic success in such a way that Em showed significantly (Tukey's test, *P* < 0.05) higher values than those of Ea and Eo, while Ec showed significantly the lowest values among the treatments (Figure 2).

### 3.3. Juvenile Survival and Growth

**Figure 3:**
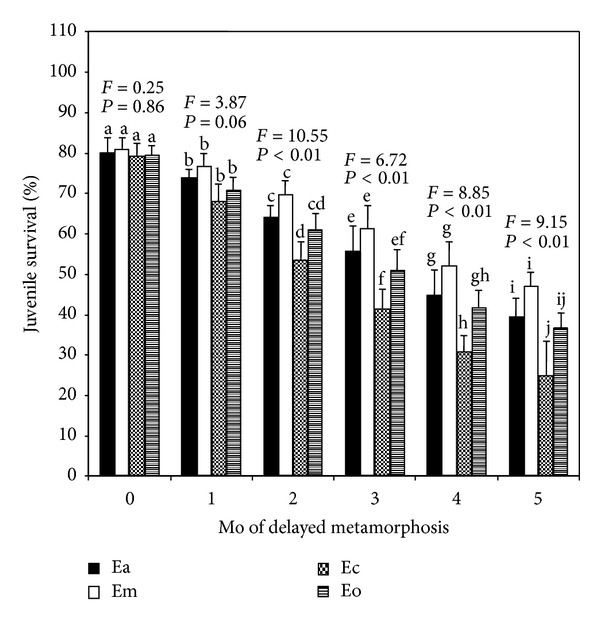
Effects of delayed metamorphosis on 1-month-old juvenile survival (%) of *Echinometra* sp. A (Ea), *E*.* mathaei* (Em), *Echinometra *sp. C (Ec), and *E*.* oblonga* (Eo). Larvae attained metamorphic competence within 24 days after fertilization were on month 0. The juveniles produced from the metamorphosis experiments were continued to rear on coralline algae for a period of 1 month. Each value represents the mean ± SD of 3 replicate experiments from each species for each delayed period. Results of one-way ANOVA (*F* value and *P* value) are presented above each set of bars. Columns with the same letter represent means that are not significantly different (Tukey's test, *P* > 0.05).

Delayed metamorphosis had a significant effect on juvenile survival: the longer that metamorphosis of competent larvae was delayed, the lower the juvenile survival of *Echinometra* spp. over the subsequent 5 months of observation (Figure 3). Larvae of all four species that attained metamorphic competence on 24 days after fertilization (0 delayed period) exhibited higher juvenile survival, although the values were significantly lower as delay period increased. In spite of this, no significant differences (Tukey's test, *P* > 0.05) were recognized in the survival rates of juveniles that obtained from the respective 0- and 1-month delayed larvae (Figure 3). In contrast, for individuals from 2- to 5-month delayed larvae of Em showed the highest values followed by Ea, Eo, and Ec in that order. Despites these discriminations in juvenile survivals, Em, Ea, and Eo did not differed significantly (Tukey's test, *P* > 0.05) from each other, while Ec differed significantly (Tukey's test, *P* < 0.05) from Em and Ea but Ec and Eo did not (Figure 3).

**Figure 4:**
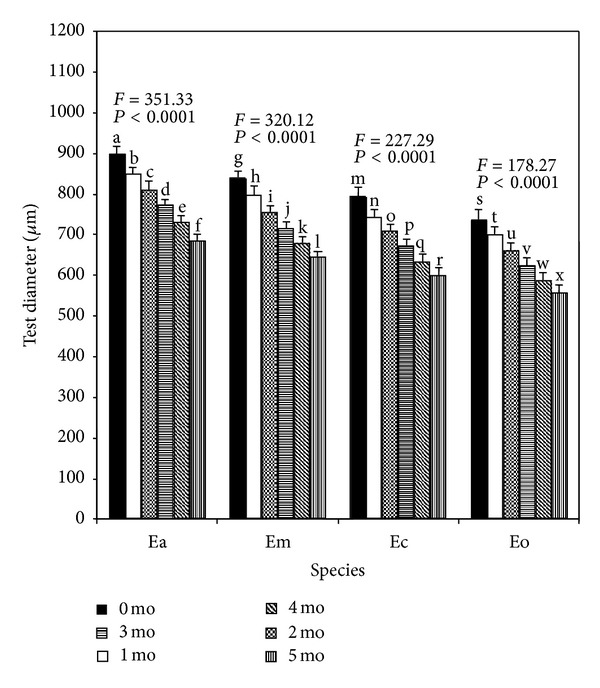
Effects of delayed metamorphosis on 1-month-old juvenile growth (test diameter in *μ*m) of *Echinometra* sp. A (Ea), *E*.* mathaei* (Em), *Echinometra *sp. C (Ec), and *E*.* oblonga* (Eo). Larvae attained metamorphic competence within 24 days after fertilization were on month 0. The juveniles produced from the metamorphosis experiments were continued to rear on coralline algae for a period of 1 month. Each value represents the mean ± SD. A total of 15 juveniles were measured for test diameter from each species with 5 individuals per replicate for each delayed period. Results of one-way ANOVA (*F* value and *P* value) are presented above each set of bars. Columns with different letters represent means that are significantly different (Tukey's test, *P* < 0.05).

**Figure 5:**
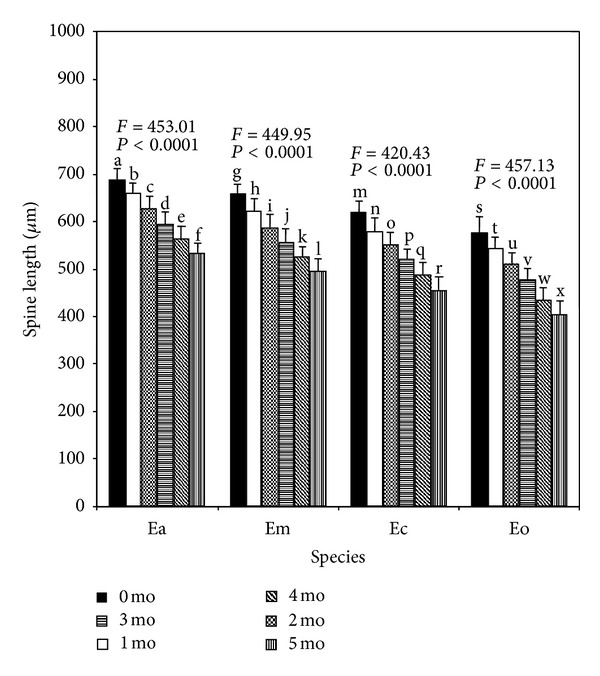
Effects of delayed metamorphosis on 1-month-old juvenile growth (spine length in *μ*m) of *Echinometra* sp. A (Ea), *E*.* mathaei* (Em), *Echinometra *sp. C (Ec), and *E*.* oblonga* (Eo). Larvae attained metamorphic competence within 24 days after fertilization were on month 0. The juveniles produced from the metamorphosis experiments were continued to rear on coralline algae for a period of 1 month. Each value represents the mean ± SD. A total of 15 juveniles were measured for spine length from each species with 5 spines per individual for each delayed period. Results of one-way ANOVA (*F* value and *P* value) are presented above each set of bars. Columns with different letters represent means that are significantly different (Tukey's test, *P* < 0.05).

The detailed growth performances in respect of test diameter and length of spines for the juveniles of the four *Echinometra* spp. at the end of 1-month culture period are summarized in Figures 4 and 5, respectively. Generally, all the growth parameters (irrespective of any delayed period) of Ea were larger followed by Em, Ec, and Eo in that order. Juveniles from larvae of all four species that metamorphosed soon after becoming competent tended to have higher growth rates than juveniles from larvae that metamorphosed after a prolonged period of competence with progressively slower growth the longer the delayed period. Therefore, duration of delayed metamorphosis had negative effects on juvenile growth rates: 1-month-old juveniles derived from the larvae that were delayed for 1, 2, 3, 4, and 5 months were significantly (Tukey's test, *P* < 0.05) smaller in respect of test diameter than control individuals induced to metamorphose on day 24 (0 delayed period) (Figure 4). Similar negative effects were also observed in spine sizes (Figure 5) of all the four species.

## 4. Discussion

The larvae of benthic marine invertebrates must typically develop for a time in the plankton before becoming capable of metamorphosing to juvenile form in order to establish bottom dwelling mode of life on their natural habitat. Although the period differs according to the developmental mode of each species, the pelagic period increases if the optimal environment and suitable settlement cue are not present. Planktonic larvae are generally not able to undergo metamorphic induction until they mature sufficiently or become “competent.” In case of sea urchins, the attainment of competence can be judged by the appearance of large juvenile rudiment and a high rate of metamorphosis [57, 68, 75]. It may occur within minutes to days for most lecithotrophic larvae or may require weeks to months for most planktotrophic larvae. Having attained competence, many pelagic larvae delay metamorphosis for variable periods of time, until they encounter a suitable substratum [2, 4, 15, 16, 20–22, 76, 77]. Planktotrophic species may remain competent for extended periods, which may vary considerably, depending primarily on food limitations [20, 29, 30, 76, 78, 79]. There has been a report that it is possible for gastropods *Cymatium nicobaricum* and *C. parthenopeum* to delay the metamorphosis for about 120 days [44]. The case of the longest delayed metamorphosis ever recorded is that of *Mediaster aequalis*, a species of starfish inhabiting the Pacific coast of North America, which was observed to delay metamorphosis of lecithotrophic larvae for about 14 month [80]. In general, a wide range of marine invertebrate larvae appears to be able to delay metamorphosis for extended periods of time. However, such researches are largely lacking in sea urchins. In this lab experiment, we observed that competent larvae of all four species of *Echinometra *were able to survive and delay metamorphosis for up to 5 month and after metamorphosis, grew into 1-month-old juveniles, even though the four species showed significant differences among themselves.

Delayed metamorphosis reduces postlarval performances in many marine invertebrates. The present study documents such effects in sea urchins in the *Echinometra mathaei* species complex. Increasing delay in larval metamorphosis had a progressively detrimental effect on postlarval growth and survival of *Echinometra* but only after an extended delay in metamorphosis. Similarly, delaying metamorphosis altered postlarval survival and growth rates in lecithotrophic abalones, barnacles, bryozoans, polychaetes, and sponges [20–22, 28, 60, 66, 79, 81]. These results contrast markedly with studies on planktotrophic polychaetes, barnacles, gastropods, nudibranchs, and echinoderms in which no adverse effects were detected by experimentally prolonging the planktonic period for days or weeks ([16, 26, 27, 82] etc.). The most widely accepted explanation for this difference is that lecithotrophs experience nutritional stress during the delay period. Delay of metamorphosis causes energy reserves to fall below the level required to metamorphose successfully and reorganize tissues in early postlarval stages [28, 66, 81]. Some reports suggest that most of the energy provisioned in the eggs is consumed during the larval stage, leaving little for the juvenile. For example, Miller [82] have estimated that a crinoid larva uses more than 80% of the energy contained in the egg during planktonic larval development and metamorphosis.

It is now well documented that marine invertebrate larvae are typically 10–100 times more sensitive to chemical and other stresses than are the juveniles and adults [83]. Such sensitivity may contribute to yearly variation in recruitment success of particular species in the field. Factors such as nutritional stress or pollutant stress that prolong larval life may also influence larval abundance by prolonging exposure to planktonic predators [84]. In addition, however, it is increasingly clear that larval experiences may affect juvenile success in more stable ways. We found that delayed metamorphosis of *Echinometra *spp. have dramatic impact on larval and juvenile survival; the longer the larval life was prolonged, the lower the percentages of larvae that recovered from various delayed period and juveniles that survived through 1 month of growth after attachment (Figures 1 and 3). These results suggest that larvae become weaker somehow as they delay their metamorphosis. Thus feeding during the period of delayed metamorphosis did not keep larvae in good condition. It appears that soon after the larvae of *Echinometra *spp. become competent, further feeding has very little impact on juvenile survival and growth even though feeding may prolong the larval life-span. In fact, we have noticed during the competent period that feeding increased larval mortality as we usually had less larvae left towards the end of the experiment. It has long been assumed that energy reserves acquired by the larvae are an important determinant of early juvenile mortality and growth [31, 85, 86].

Influence of delayed metamorphosis on juvenile performances was reported even for planktotrophic (feeding) larvae of polychaetes that were fed sufficiently throughout larval period [20, 62]. This suggests that nutritional stress is an insufficient explanation for the influence of extended larval life on postmetamorphic performance [20, 21, 28]. It has been hypothesized that extreme delays in metamorphosis may affect postlarval fitness by compromising the larva's preparations for postlarval feeding and growth and, consequently, postlarval juvenile performance [21]. If larvae are forced to postpone metamorphosis, juveniles of some species show higher postsettlement mortality [18, 62, 76, 79], greater sensitivity to physical stress [16], or slower rates of juvenile growth or development [27, 29, 30, 62, 66]. In this study, we found that delayed metamorphosis alone had a strong negative effect on juvenile growth, as 1-month-old juveniles resulting from larvae that were delayed for 1, 2, 3, 4, and 5 months were significantly smaller than controls triggered to metamorphose on month 0. Thus, it is becoming apparent that events experienced by larvae can substantially influence juvenile performance.

Despite the adverse effects of delaying metamorphosis on growth parameters, the present study demonstrated that competent larvae of all four species were able to survive up to 5 month-, and after metamorphosis, grew into 1 month- old juveniles in lab condition. These extended larval lifetimes and competent periods imply the potential for dispersal for up to several months in *Echinometra* larvae. The extent to which the duration of the pelagic period varies in field and how distributions are affected by delaying metamorphosis and other biotic and abiotic factors are unknown. These data, although laboratory limited, are the first contribution from the widespread tropical sea urchins like *Echinometra*. Results presented here for *Echinometra* spp., though, are from laboratory experiments and thus indicate possible but perhaps not actual pelagic periods in the field. Dispersal of larvae has long-term consequences of gene flow, geographic range of species, speciation, extinction, and species longevity, although feeding larvae may also be overdispersed [78, 87]. Speciose marine taxa like echinoderms, mollusks, and polychaete annelids have large number of species with larvae that show high dispersal potential [15, 33]. Coupled with this high dispersal is the fact that the World's oceans are not extremely subdivided. As a result, there are very few absolute barriers to dispersal or gene flow in the sea (see Lessios [88], for one of the rare major exceptions, the Isthmus of Panama). The combination of high dispersal and incomplete geographic barriers often leads to huge ranges for many marine species. Particularly in the tropical Pacific, species range often span over 10,000 km [89–91]. Across this range, cumulative population size can be very large, and there may be substantial population structure in some whales, urchins, and billfish [92–94]. For *Echinometra* spp., as far as data showed, the long planktonic life seems to be related to dispersal and genetic interchange in a wide geographical range of Indo-Pacific region.

**Figure 6:**
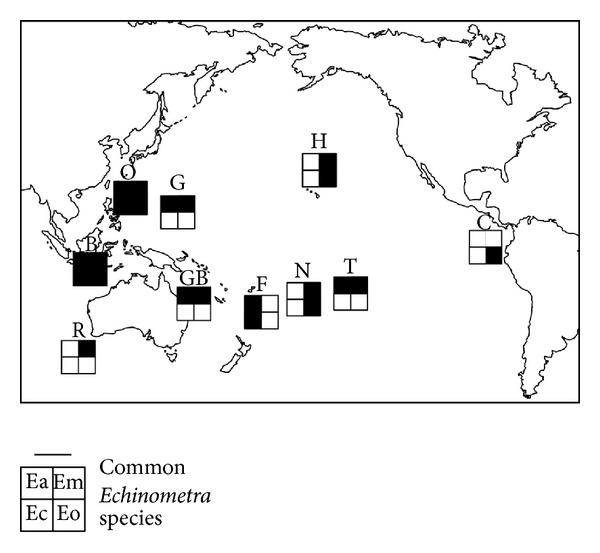
Distribution patterns of common *Echinometra* spp. on the Pacific reef (after Palumbi [49]). Species are designated according to the published documents [8, 49–58]: Ea, *Echinometra* sp. A; Em, *E. mathaei*; Ec, *Echinometra *sp. C; and Eo, *E. oblonga*. Localities are C, Isla del Coco, Costa Rica; H, Hawaiian Islands; G, Guam; O, Okinawa; B, Bali; GB, Great Barrier Reef; R, Rottnest Is.; F, Fiji; N, Niue; and T, Tahiti.

All of the four species of *Echinometra *occur on reefs in Okinawa and Indonesia. It is more common, however, to find only two sympatric species in island archipelagoes of the central/western pacific. At the edge of the tropical pacific, *Echinometra* species are often found alone (e.g., *E. oblonga* in Cocos Is., Costa Rica and *E. mathaei* at Rottnest Is., SW Australia) [49]. Until now, there have been no detailed information of the distribution patterns of *Echinometra*, but from the recently incomplete survey done by Palumbi [49], it is clear that discontinuities of species ranges are common for Pacific *Echinometra* (Figure 6). For example, *E. mathaei* and *E. oblonga* are found together in Hawaii and in Niue. In Fiji, 1300 km to the west of Niue, there are also two species of *Echinometra* but not *E. mathaei* and *E. oblonga*. Instead, *E.* sp. A and *E*. sp. C are common (Figure 6). To the east of Niue, in the Society Islands, only *E. mathaei* and *E.* sp. A are common. As a second example, *E. mathaei* and *E.* sp. A are the common calm-water species in Guam and Papua New Guinea. In Palau (1300 km SW of Guam) *E.* sp. C is the commonest species of *Echinometra* with *E.* sp. A occurring rarely. Highly realized dispersal in the Pacific is suggested by the discovery of indistinguishable mtDNA sequences in individuals collected from distant localities [49]. For *E.* sp. A, identical sequences were seen in the individuals collected from Guam and Bali (4000 km distant), Fiji and Papua New Guinea (4500 km), and Okinawa and Guam (2600 km). The observation of indistinguishable mtDNA sequences in geographically distant localities is common for marine species with high dispersal potential [95–97]. If a reef can support populations of several species of *Echinometra*, and *Echinometra* species have high dispersal potential from reef to reef, why are not all four species found in all available reef habitats? One potential explanation for the heterogeneous distribution of species in the Pacific is that local populations are founded on archipelagoes largely by chance. If these founder events are relatively rare, there may not have been enough time since these species formed for the colonization of all available reefs in the Pacific by all species [49]. Micronesian ostracods also show this pattern [98], with distinct heterogeneity between atolls in species composition but no clear relationship between species similarity and distance between atolls.

Geographic patterns of mtDNA haplotypes parallel patterns of species ranges are large but distributions are heterogeneous. In case of *E.* sp. A, some islands that are separated by 2600 km show very different frequencies of mitochondrial haplotypes (e.g., Guam and Okinawa) despite sharing different individual haplotypes. However, this geographic subdivision is not always observed. In some cases, very similar gene frequencies are found at localities separated by 4500 km (e.g., Fiji and Papua New Guinea) [49]. Thus the high dispersal potential of *Echinometra* is reflected in wide distribution of mtDNA genotypes and high similarity between some populations, but this potential does not result in genetic homogeneity of populations across even moderate spatial scales [49]. For sea urchins in the genus *Echinometra*, there is strong mtDNA differentiation among populations within all four species that have been observed [49]. However, comparison of gene flow patterns for the four species reveals no concordance. Each species shows mtDNA regionalization (similar mtDNA haplotype frequencies across a range of few thousand kilometers), the boundaries of these mtDNA regions in each species do not coincide, for example, populations of *E. oblonga* for two major clusters of mtDNA relatedness. One cluster is in the western Pacific and includes Papua New Guinea and Okinawa, whereas as the other cluster is to the east, at the periphery of the Indo-West Pacific including Hawaii and Niue [96]. By contrast, in *E. mathaei*, there is a region in the Northern Hemisphere including Hawaii, Guam, and Okinawa and an isolated southern region that includes Australia, Niue, and Tahiti. Lack of concordance in geographic genetic differentiation suggests that gene flow in these two species is determined by different factors, even though the species are very closely related and have very similar life histories and ecologies and thus gene flow is strongly subject to unpredictable dispersal during EI Niño events [96].

Overall, delayed larvae of Em from the present study showed significantly higher larval survival, metamorphosis, and juvenile survival than Ea and Eo, while Ec showed the lowest values in these performances. Em is the most abundant and widespread of these species, occurring across a huge range from Hawaii and Tahiti to East Africa and the Persian Gulf [49, 50, 72, 96], while Ec probably has the most restricted distribution, being known only in the western Pacific [49, 58, 70, 72, 99]. Consequently, differences in the ability to delay metamorphosis may be related to differences in distribution of *Echinometra* spp. throughout the Indo-Pacific regions.
